# Response and Failure of Pillar–Backfill Composite Materials Under Cyclic Loading: The Role of Pillar Width

**DOI:** 10.3390/ma19081625

**Published:** 2026-04-17

**Authors:** Qinglin Shan, Changrui Shao, Hengjie Luan, Sunhao Zhang, Chuming Pang, Yujing Jiang, Lujie Wang

**Affiliations:** 1 State Key Laboratory of Disaster Prevention and Ecology Protection in Open-Pit Coal Mines, Shandong University of Science and Technology, Qingdao 266590, China; skd995946@sdust.edu.cn (Q.S.); shaochangrui@sdust.edu.cn (C.S.); 201981010010@sdust.edu.cn (C.P.); jiang@nagasaki-u.ac.jp (Y.J.); 19863760029@163.com (L.W.); 2 Graduate School of Engineering, Nagasaki University, Nagasaki 852-8521, Japan; bb52222191@ms.nagasaki-u.ac.jp; 3 Academician (Expert) Workstation, Inner Mongolia Shanghaimiao Mining Co., Ltd., Ordos 016299, China

**Keywords:** pillar–backfill composite materials (PBCM), low-frequency disturbance, acoustic emission (AE), digital image correlation (DIC), failure mode

## Abstract

In the deep mining of metal mines, the stability of pillar–backfill composite materials (PBCMs) under cyclic loading is crucial for preventing dynamic disasters in goafs. Although previous studies have extensively investigated backfill materials under static loading, the damage evolution mechanism of PBCM under cyclic disturbance—particularly the coupled effects of pillar width and disturbance amplitude—remains insufficiently understood. To address this gap, this study explored the mechanical properties and damage evolution of PBCM under cyclic loading using an indoor testing system. Tests were conducted on composite specimens with varying pillar widths (6, 9, 12, 15 mm) and disturbance amplitudes (3, 4, 5 MPa), combined with acoustic emission (AE), digital image correlation (DIC), and scanning electron microscopy (SEM). Results show that wide-pillar specimens (≥12 mm) exhibit significantly improved bearing strength and deformation modulus, with increases of nearly 90% and over 40%, respectively, compared to narrow-pillar specimens. Notably, wide pillars maintain over 95% strength stability even under 5 MPa cyclic disturbances. Narrow pillars are prone to localized damage concentration with high-frequency AE signals and shear failure, while wide pillars exhibit uniform damage development. Failure morphology confirms that pillar size dictates failure mode: narrow pillars undergo sudden through failure, whereas wide pillars display progressive composite failure, with fewer damage-induced cavities and directional crack propagation along maximum shear stress. These findings provide a theoretical basis for stope structure optimization and dynamic disaster prevention in deep mines.

## 1. Introduction

Cemented backfill mining technology has become a key technology for achieving safe and efficient mining in modern metal mines by organically combining cemented backfill with reserved pillars to form a pillar–backfill composite material (PBCM) [1,2,3]. This technology can not only effectively control the deformation of surrounding rock in goafs but can also significantly improve the recovery rate of mineral resources [4]. As the core load-bearing body of the mine mining system, the mechanical properties of PBCM directly determine the overall stability of the stope. However, in practical engineering, due to the complexity of geological structures and the dynamics of mining activities, PBCM is often in an inhomogeneous stress field. Especially under deep mining conditions, high in situ stress and complex geological environments make the mechanical response of PBCM more complicated [5,6,7]. Therefore, an in-depth exploration of the mechanical properties and evolution laws of PBCM is of great theoretical significance and engineering value for optimizing stope structure design and ensuring safe mine production.

**Figure 1:**
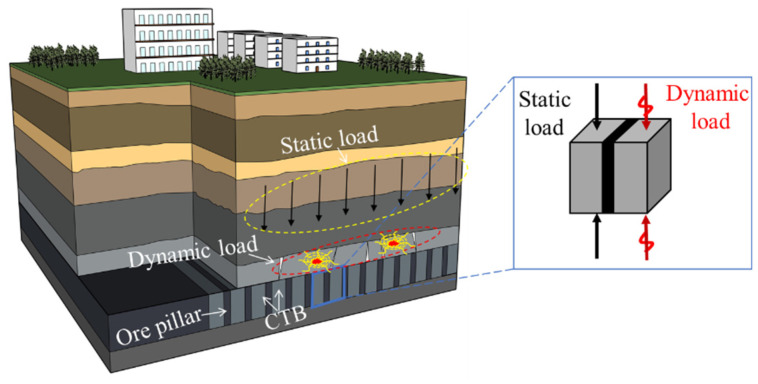
Goaf structure and test model diagram.

In the field of research on the mechanical properties of the PBCM, scholars at home and abroad have made significant progress [8,9,10,11,12]. Among them, Wang et al. [13] studied the mechanical properties and macroscopic fracture characteristics of the composite materials through uniaxial compression, and discussed the crack propagation and energy evolution laws through simulation. Zhao et al. [14] conducted uniaxial compression tests on composite materials samples with different backfill strengths, and analyzed the influence of changes in backfill strength on the mechanical properties of the composite materials. Cui et al. [15] used the digital image correlation (DIC) method to study the deformation characteristics and damage evolution process of six types of composite materials under uniaxial compression conditions. As the main load-bearing unit, changes in the pillar’s width will significantly alter the overall mechanical behavior of the composite materials [16]. Wu et al. [17] summarized the pillar stability analysis methods by mechanically dissecting the pillar formation process and explored the influence of pillar size on their mechanical response and stability. Sherizadeh et al. [18] used 3DEC numerical simulation to analyze the influence of pillar size on roof stability. The above studies systematically revealed the evolution laws of mechanical properties and the failure mode characteristics of backfill, especially the key regulatory role of pillar width changes in the overall strength, deformation characteristics, and damage evolution process of backfill. However, in engineering practice, PBCM often bears cyclic loading, such as roof breaking and blasting vibrations (Figure 1), and the progressive damage accumulation caused by such loading is more significant than that under static conditions, leading to the accelerated deterioration of load-bearing performance. Although the research on the mechanical behavior of PBCM under static loading is relatively complete, the research on the synergistic load-bearing mechanism under cyclic loading is still insufficient, which directly affects the accurate evaluation of the long-term stability of backfill in deep mines.

During the process of mine exploitation, pillar–backfill composite materials (PBCM) continuously bear periodic loading. Such cyclic stress will induce the progressive accumulation of fatigue damage inside the material, thereby significantly affecting the long-term stability of the structure [19,20,21]. Zhou et al. [22] conducted indoor cyclic loading tests on rock samples and analyzed the relationship between damage variables and strain rates under different peak strength ratios in the post-peak stage. Li et al. [23] analyzed the crack propagation process and mechanical properties of rock samples under fatigue loading based on acoustic emission technology, b-value, and the evolution characteristics of AF-RA distribution. Gan et al. [24] performed uniaxial compression tests on backfill samples after cyclic loading, studying the impacts of loading gradient, loading rate, and loading time on their mechanical properties and damage characteristics. Numerous studies have shown that cyclic loading significantly deteriorates the stability of cemented tailings backfill. However, existing research has mainly focused on the mechanical properties and damage evolution of homogeneous materials, while the synergistic load-bearing mechanism of the pillar–backfill composite structure—a heterogeneous material widely applied in mining engineering—under cyclic loading remains poorly understood.

Based on the above background, this study employs a cyclic loading test system to investigate the mechanical response characteristics of pillar–backfill composite structure (PBCM) specimens. The study focuses on examining the effects of disturbance amplitude and pillar width on the strength and deformation behavior of the specimens. By integrating acoustic emission characteristic parameters with the evolution of digital speckle strain fields, the damage development mechanism of the specimens is revealed. Additionally, scanning electron microscopy (SEM) is utilized to observe microscopic features such as microcrack propagation and mineral fragmentation. The research findings provide a theoretical basis and technical support for the optimal design of stope structures and the prevention and control of dynamic disasters in deep mines.

## 2. Sample Preparation and Test Plan

### 2.1. Structure Material Composition of PBCM

The pillar–backfill composite samples used in this test consist of pillars and backfill materials. The pillar raw materials were taken from an iron mine in Shandong Province, while the backfill materials were prepared by using tailings as aggregates, mixed with sulphoaluminate cement and tap water. According to the X-ray diffraction (XRD) (Dandong Tongda Technology Co., Ltd., Dandong, China) analysis in Wang’s experiment, the main mineral compositions of the pillars are: grunerite (51.1%), hematite (14.3%), magnetite (22.5%), and quartz (8.9%); the main mineral compositions of the tailings are: silicon oxide (67.1%), granite (18.8%), and magnetite (8.2%) [25]. Referencing the actual filling material ratio used in the mine and combining it with previous studies [13], the test ultimately confirmed the ratio scheme of 76% slurry concentration and a 1:4 cement-to-sand ratio (cement:tailing sand:water = 1:4:1.58). This ratio ensures good workability of the slurry while meeting the dual requirements of material strength and economic feasibility in engineering practice.

### 2.2. Sample Preparation Process

**Figure 2:**
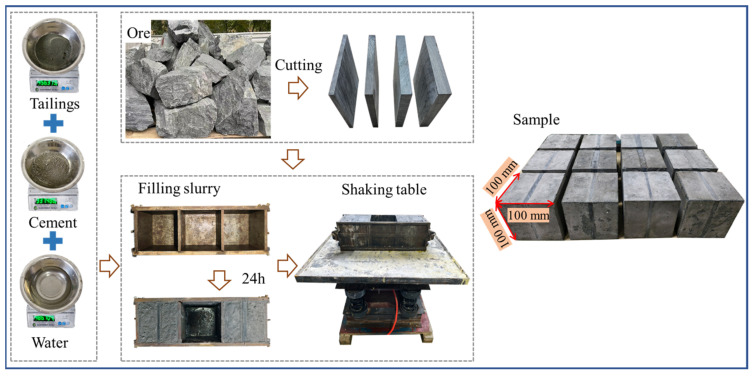
Preparation process of PBCM.

The preparation process of the PBCM samples involves three key steps, as shown in Figure 2. Firstly, the original ore is mechanically processed into standard-sized samples. The pillars are uniformly maintained at a length and height of 100 mm, while their widths are machined into four specifications (6 mm, 9 mm, 12 mm, and 15 mm) according to the test requirements. The surfaces of the pillars are precision-ground to meet the test accuracy requirements. Secondly, backfill slurry is prepared at a cement-to-tailings ratio of 1:4. After fully mixing and stirring tailings, cement, and water, the mixture is poured into a standard cubic mold of 100 mm × 100 mm × 100 mm, and vibrated compactly on a shaking table to eliminate internal air bubbles. Finally, the samples are demolded after 48 h of initial curing, then transferred to a standard curing environment (temperature 20 ± 2 °C, relative humidity ≥ 95%) for continuous curing for 28 days. After the curing period, the two end faces of the samples are finely ground to ensure that the flatness of the contact surfaces meets the test specification requirements.

### 2.3. Test Equipment

**Figure 3:**
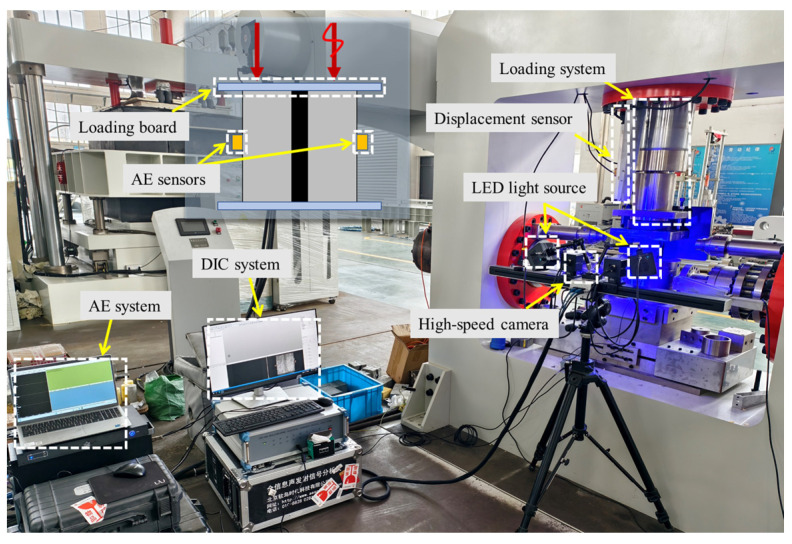
Test equipment diagram.

The configuration of the test loading and monitoring system is shown in Figure 3. The loading equipment is the rock and soil mass multi-field coupling dynamic and static characteristic test system (Qingdao Qiankunxing Intelligent Technology Co., Ltd., Qingdao, China) from the College of Energy and Mining Engineering, Shandong University of Science and Technology. This system is equipped with a Germanjet1700210200 high-precision displacement sensor (with an accuracy of 0.005% F.S) for real-time monitoring of the axial displacement of the sample. To comprehensively monitor the sample failure process, an acoustic emission monitoring system and a digital image correlation measurement system are configured simultaneously. The acoustic emission monitoring (Beijing Softland Times Technology Co., Ltd., Beijing, China) adopts the DS5 acquisition system with the RS-55A probe. The threshold and sampling rate of acoustic emission acquisition are set to 40 dB and 10 MHz, respectively. Prior to the test, pencil lead break tests were performed for sensor calibration, with each sensor tested three times and localization error controlled within ±5 mm. In this study, a dual-probe symmetric arrangement scheme is adopted, where two acoustic emission probes are accurately installed at the central positions on both sides of the sample. The probes were fixed with adhesive tape using Vaseline as a coupling agent to achieve the comprehensive collection and accurate measurement of acoustic emission signals.

Meanwhile, the XTDIC (XTOP 3D Technology (Xi’an) Co., Ltd., Xi’an, China) three-dimensional digital speckle dynamic measurement system is used to monitor the surface deformation field of the sample. This system consists of a CCD high-speed camera, a high-brightness LED cold light source, and a professional image processing workstation, and realizes real-time dynamic observation of the surface displacement field of the sample through a non-contact measurement method. Among them, the size of the speckle sub-region is 21 × 21 pixels, the step length is 15 pixels, and the pixel of the CCD high-speed camera is 2048 × 1536. The ZEM18 desktop scanning electron microscope (Anhui ZEION Technology Co., Ltd., Tongling, China) is used to study the microstructure of the sample after failure.

### 2.4. Test Scheme

Uniaxial compression tests were carried out on the samples of the pillar–backfill composite materials, and the uniaxial compressive strengths of the samples with different pillar widths (6 mm, 9 mm, 12 mm, and 15 mm) were measured, which are 13.21 MPa, 14.86 MPa, 17.11 MPa, and 20.33 MPa, respectively. Based on these values, the initial disturbance value σ was determined [26].

**Figure 4:**
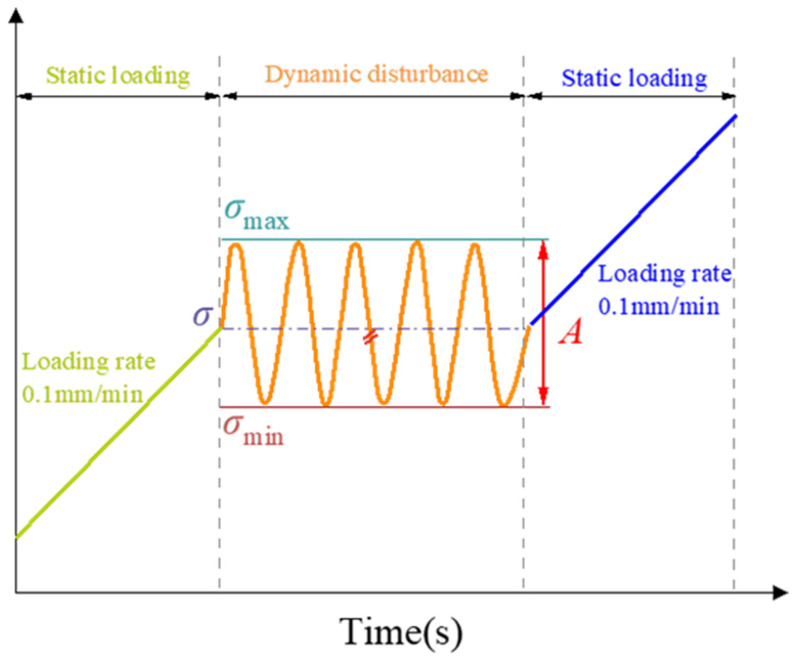
Loading scheme.

The loading scheme is shown in Figure 4. Firstly, in the static loading stage, the displacement control mode is adopted to apply the load to the predetermined static load σ at a loading rate of 0.1 mm/min. In the cyclic disturbance stage, with the static load σ as the reference stress, a sinusoidal cyclic load with an amplitude A is applied. According to the on-site working conditions, the loading frequency is 5 Hz and the number of cycles is 1000. Finally, in the failure loading stage, the pressure continues to be applied at a loading rate of 0.1 mm/min until the sample is destroyed.

The sample number adopts the naming rule of “pillar width–disturbance amplitude”. For example, 6-2 represents a sample with a pillar width of 6 mm and a disturbance amplitude of 4 MPa. The specific test parameters are shown in Table 1.

## 3. Analysis of Test Results

### 3.1. Stress–Strain Curve Characteristics

**Figure 5:**
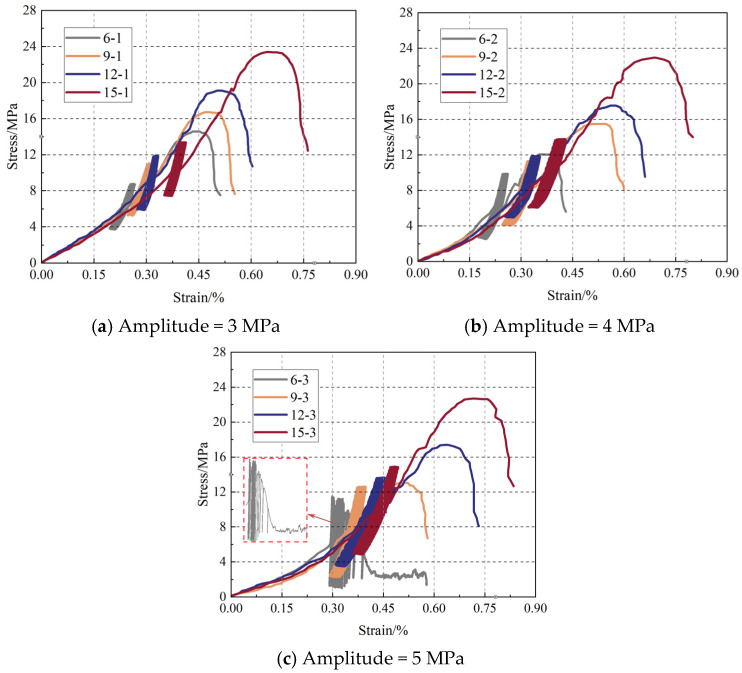
Stress–strain curve of PBCM sample under cyclic load.

Figure 5 shows the cyclic load stress–strain curves of the pillar–backfill composite materials under different disturbance amplitudes and pillar widths. Through comparative analysis of the 12 groups of curves, it can be observed that the mechanical response of the composite materials under cyclic loading presents obvious three-stage characteristics: the initial static load stage, the cyclic disturbance stage, and the final failure stage. Among them, disturbance parameters and pillar sizes have significant impacts on the deformation characteristics of each stage.

In the initial static load stage, the stress–strain curves of all samples show significant nonlinear compaction characteristics, and the overall change trend is highly consistent. After entering the cyclic disturbance stage, the curves with different parameter combinations show significant differences. It is worth noting that the sample with a pillar width of 6 mm can only complete 90 cycles under a disturbance amplitude of 5 MPa, and its hysteresis loop shows an obvious “fan-shaped expansion” feature in the later stage, indicating that severe damage accumulation has occurred inside the structure at this time. In contrast, the 15 mm pillar sample can still maintain a relatively regular hysteresis loop shape under the same disturbance conditions, showing better cyclic stability.

In the final failure stage, as the axial stress continues to increase, the microcracks inside the pillar gradually expand and connect with each other, resulting in a significant decrease in its bearing capacity. At this time, the backfill materials begins to play a synergistic bearing role, forming a new bearing system together with the residual pillar to maintain the overall stability of the sample. It is noteworthy that the stress–strain curves of some samples show a short stress platform after reaching the initial strength extreme value, and then the curves rise again. This feature fully reveals the synergistic bearing mechanism between the pillar and the backfill materials. When the load continues to increase to the bearing limit of the backfill materials, the sample finally undergoes overall failure.

### 3.2. Strength Deformation Characteristics

**Figure 6:**
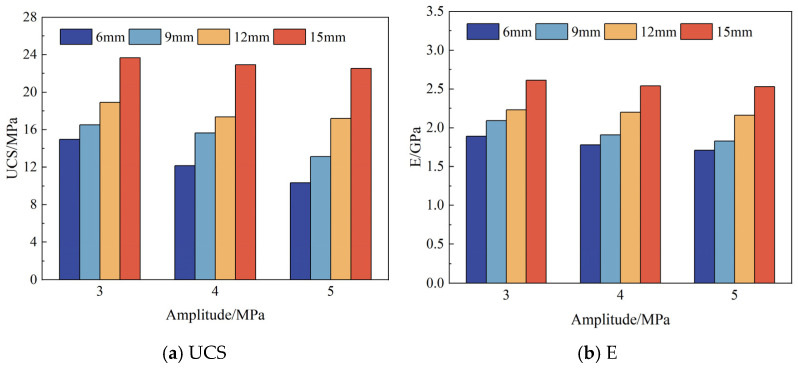
Changes in UCS and E of PBCM samples under cyclic load.

Figure 6 and Table 2 show the UCS and E of PBCM samples under cyclic loading. The elastic modulus E was determined by linear fitting of the linear elastic portion of the stress–strain curve from the first loading cycle, using a consistent strain interval (10–40% of peak strength) across all specimens. Both the pillar width and disturbance amplitude have significant impacts on the uniaxial compressive strength (UCS) and elastic modulus (E) of the samples. As the pillar width increases, both UCS and E show an upward trend. For example, when the disturbance amplitude is 3 MPa, as the pillar width increases from 6 mm to 15 mm, the UCS of the sample significantly increases from 12.15 MPa to 22.92 MPa, with an increase rate of 88.6%; correspondingly, E increases from 1.78 GPa to 2.54 GPa, with an increase rate of 42.7%. This gradient change in mechanical properties indicates that increasing the pillar width can effectively improve its bearing capacity and deformation resistance.

At the same time, the increase in disturbance amplitude will lead to a decrease in the UCS and E of the sample. Taking the 6 mm pillar sample as an example, when the disturbance amplitude increases from 3 MPa to 5 MPa, the UCS decreases from 14.96 MPa to 10.34 MPa, with a decrease rate of 30.9%, and E decreases from 1.89 GPa to 1.71 GPa, with a decrease rate of 9.5%. This phenomenon indicates that dynamic disturbance has an obvious deteriorating effect on the mechanical properties of the sample, which is attributed to the internal microcrack propagation or cumulative damage effect caused by the disturbance load. It is worth noting that samples with larger pillar widths are less sensitive to the disturbance amplitude. For instance, when the disturbance amplitude increases from 3 MPa to 5 MPa, the UCS of the 15 mm pillar sample only decreases by 4.8%, and the decrease in E is 3.1%, which is much lower than the deterioration degree of the 6 mm pillar sample. This is because the larger-sized pillar can avoid the rapid damage accumulation caused by local stress concentration, indicating that increasing the pillar size can alleviate the adverse effects of dynamic disturbance to a certain extent.

### 3.3. “AE DIC” Characteristics

**Figure 7:**
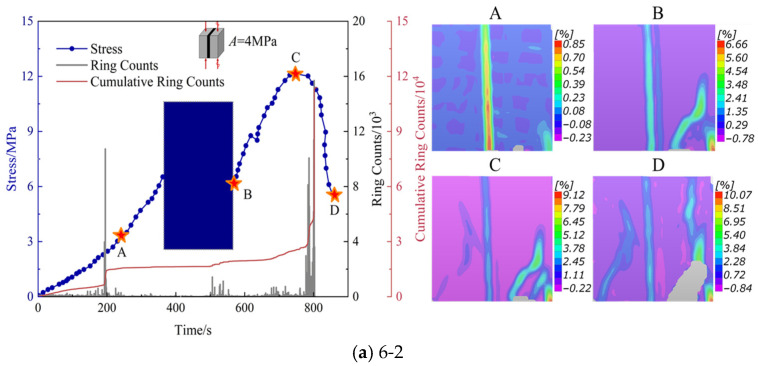

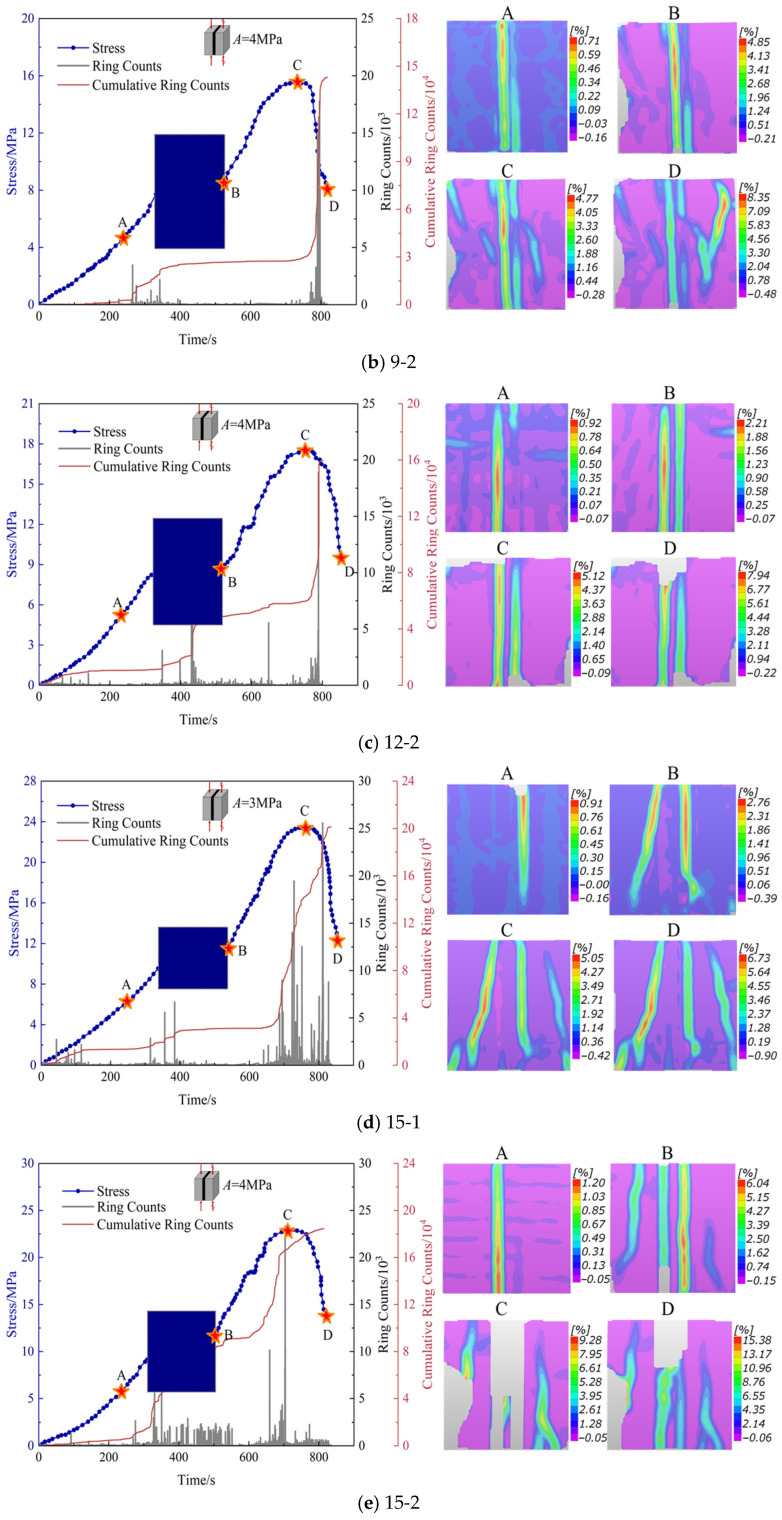

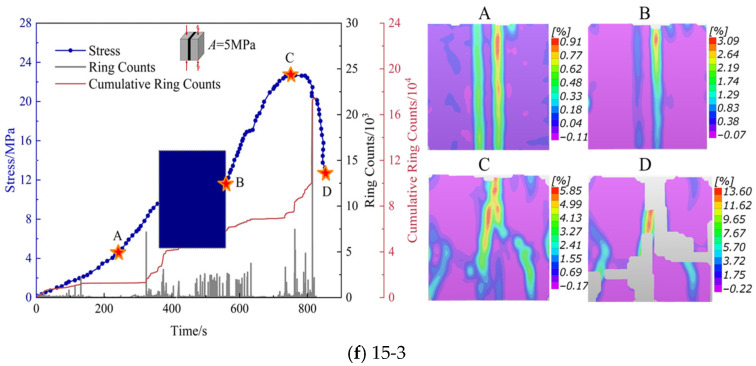
AE counts and DIC evolution of PBCM.

Figure 7 illustrates the evolution law of acoustic emission energy and the distribution characteristics of digital image correlation (DIC) strain fields for PBCM samples under cyclic loading. Six samples, namely 6-2, 9-2, 12-2, 15-1, 15-2, and 15-3, were selected for analysis. Four characteristic points (Point A: end of compaction stage; Point B: end of cyclic loading stage; Point C: peak point; Point D: final failure point) were chosen from the stress–strain curves to compare the changes in the speckle strain fields.

Through comparative analysis of Figure 7a–c,e, it can be revealed that the pillar width has a significant impact on the damage evolution characteristics under cyclic loading. During the static loading stage, samples with narrower pillars (6 mm and 9 mm) exhibited more active acoustic emission signals, indicating the rapid initiation and propagation of internal microcracks, while those with wider pillars (12 mm and 15 mm) showed weaker acoustic emission activities. At this time, the DIC strain fields indicated that the strain concentration areas of all samples was mainly distributed in the middle of the pillars, showing localization characteristics. After entering the cyclic disturbance stage, the frequency of acoustic emission activities in narrow pillar samples decreased, but sudden high-energy events increased, reflecting that their internal damage entered a stage of unstable expansion. Meanwhile, the strain concentration areas gradually expanded to both ends and formed obvious shear bands. In contrast, the acoustic emission signals of wide pillar samples continued to strengthen, the cumulative energy linearly increased, and the strain fields remained relatively uniformly distributed, with only local areas showing an increase in strain gradient. In the failure stage, the cumulative ringdown counts of samples with pillar widths of 6 mm, 9 mm, 12 mm, and 15 mm were 119,564, 143,755, 161,543, and 189,341, with increases of 20.2%, 12.4%, and 17.2%, respectively. At the same time, the acoustic emission activities of narrow pillar samples (6 mm and 9 mm) continued to strengthen, and the strain fields showed multi-region coordinated deformation, eventually forming a network crack system. For wide pillar samples (12 mm and 15 mm), a significant sudden increase in acoustic emission ringdown counts occurred before macroscopic fracture, accompanied by the penetration of strain concentration areas.

Further analysis of the influence of disturbance amplitude (Figure 7d–f) was conducted using 15 mm pillars as an example. As the disturbance amplitude increased from 3 MPa to 5 MPa, the cumulative acoustic emission energy of the samples increased significantly, especially in the second stage, where acoustic emission activities under a high disturbance amplitude (5 MPa) were more intense. With the increase in disturbance amplitude, the cumulative ringdown counts at sample failure were 203,082, 189,341, and 181,563, with decreases of 6.73% and 4.11% respectively. This indicates that high disturbance loads will accelerate damage concentration, inhibit the progressive expansion of microcracks, and cause the material to fail at a lower cumulative ringdown count. The DIC strain fields showed that under a low disturbance amplitude (3 MPa), the strain concentration areas were mainly distributed at the ends of the pillars, while under a high disturbance amplitude (5 MPa), the strain concentration phenomenon expanded to the interior of the pillars, with local strain values increasing by 30–45%.

A comprehensive comparison shows that pillar width and disturbance amplitude jointly affect the failure mode. Samples with narrow pillars (6 mm and 9 mm) exhibit more uniform damage evolution, while those with wide pillars (12 mm and 15 mm) show stronger anti-disturbance ability but are prone to localized shear failure under cyclic disturbance, with acoustic emission activities concentrated in the pre-failure stage. In addition, the increase in disturbance amplitude will significantly accelerate the rate of damage accumulation inside the pillars, expand the range of strain concentration, and ultimately reduce their overall stability.

### 3.4. Macroscopic Failure Characteristics

**Figure 8:**
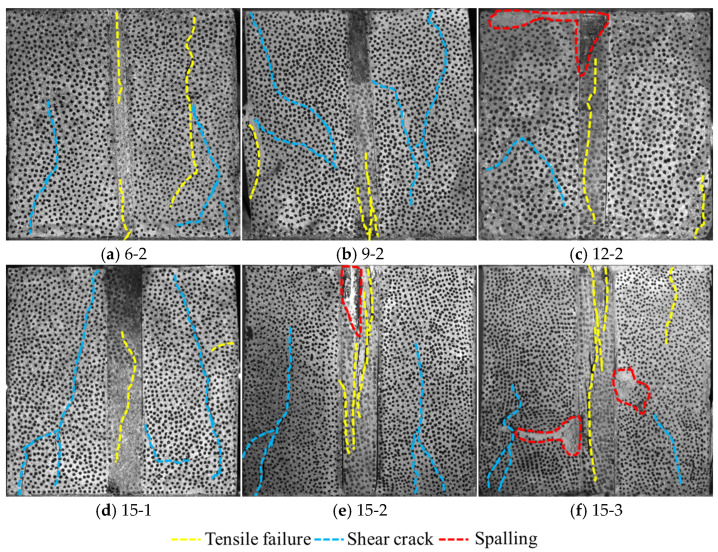
Macroscopic damage characteristics of PBCM samples.

Figure 8 shows the macroscopic damage characteristics of the composite material samples. The annotations highlight the three typical failure characteristics of the specimens: tensile cracks, shear cracks, and spalling. From the perspective of macroscopic failure morphology, samples under different pillar widths and disturbance amplitudes exhibit distinct differences in failure modes. During the failure process of the 6 mm pillar sample (6-2), shear cracks mainly develop in the left backfill, while both dendritic shear cracks and tensile cracks appear in the right backfill, with two significant tensile cracks forming inside the pillar. As the pillar width increases to 9 mm (9-2), the failure characteristics of the sample change: dendritic shear cracks occur in both the left and right backfills, with additional tensile cracks in the left backfill, and the pillar failure is concentrated in the tensile crack at the lower end. The failure of the 12 mm pillar sample (12-2) is relatively localized: shear cracks and upper-end spalling appear in the left backfill, only short tensile cracks form in the right backfill, and spalling occurs at the upper end of the pillar. The 15 mm wide pillar sample (15-2) presents a more complex failure morphology: dendritic shear cracks develop in both side backfills, and the pillar not only has upper-end spalling but also generates multiple long tensile cracks inside.

The disturbance amplitude has a significant impact on the failure characteristics of the 15 mm pillar samples. Under the low amplitude of 3 MPa (15-1), only dendritic shear cracks form in the left backfill of the sample, two shear cracks appear on the right side, and a single tensile crack is generated inside the pillar. When the amplitude increases to 4 MPa (15-2), the damage degree intensifies significantly: dendritic shear cracks develop in both side backfills, and the pillar has upper-end spalling with multiple long tensile cracks. When the amplitude further increases to 5 MPa (15-3), the sample suffers the most severe damage: the left backfill has both dendritic shear cracks and spalling, the right backfill generates short shear cracks, tensile cracks, and spalling, and multiple interlaced tensile cracks develop inside the pillar. Under the low amplitude of 3 MPa, the energy input per cycle is limited, mainly leading to the stable propagation of interface microcracks and local shear failure. When the amplitude increases to 4 MPa, the energy input per cycle exceeds the material’s critical value, triggering accelerated crack propagation, which manifests as a significant increase in the number and length of cracks. Under the high amplitude load of 5 MPa, the energy input far exceeds the material’s fracture toughness, resulting in rapid crack penetration and brittle fracture of the material, with the failure mode showing crushing characteristics. It is worth noting that as the pillar width increases, the energy storage capacity of the sample enhances, enabling it to absorb more energy before failure. This allows wide pillar samples to maintain a certain degree of integrity under high-amplitude loads, with their failure process showing progressive characteristics.

### 3.5. SEM Image Feature Analysis

**Figure 9:**
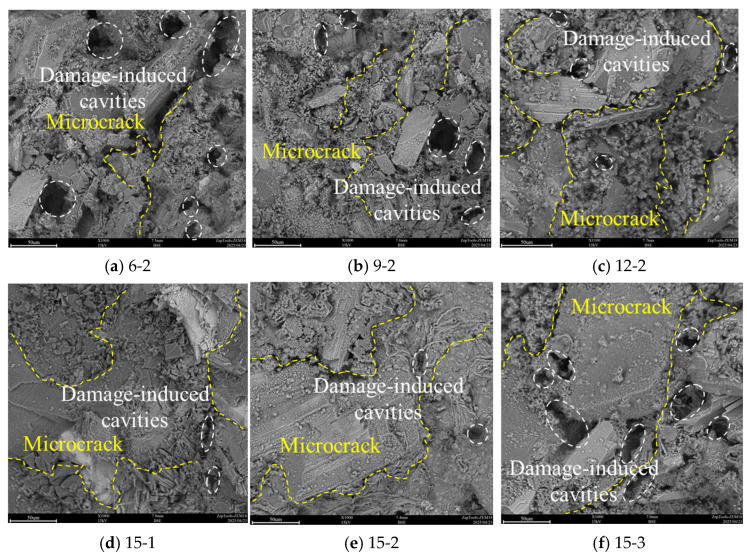
SEM scanning image of the backfill.

To further analyze the damage characteristics of PBCM samples under cyclic loading, the ZEM18 high-resolution desktop scanning electron microscope (SEM) was employed to characterize the microtopography of the fracture surfaces/damaged areas of the samples. These damage-induced cavities, which form during cyclic loading, exhibit irregular shapes and rough surfaces, and are often connected to microcrack networks. By observing microfeatures such as microcrack propagation and mineral fragmentation, SEM technology provides a key basis for establishing a correlation model between materials damage and macroscopic mechanical properties [27,28,29]. Through analyzing the distribution patterns of damage-induced cavities and fractures, it is found that both pillar width and disturbance amplitude jointly regulate the failure mode of the material, as shown in Figure 9. In samples with narrow pillars (6–9 mm), a large number of damage-induced cavities and intersecting fractures appear in the backfill part, mainly resulting from local stress concentration caused by the end constraint effect. Under cyclic loading, the edge areas of the backfill are subjected to repeated normal stress, which promotes intergranular fracture and forms damage-induced cavities of varying sizes. As the pillar width increases to 12–15 mm, the stress field distribution tends to be uniform, the number of damage-induced cavities decreases significantly, and the fracture morphology changes from intersecting to long fractures extending along the direction of maximum shear stress. This size effect reflects the optimization of the load transfer path by the geometric constraint of the pillar.

The influence of disturbance amplitude on damage characteristics exhibits an obvious threshold effect. When the pillar width is fixed at 15 mm, the backfill suffers the least damage under the 4 MPa disturbance condition, with only a small number of small damage-induced cavities and regular long fractures appearing, indicating that the energy input and material damage dissipation reach a dynamic balance at this point. When the disturbance increases to 5 MPa, the number of damage-induced cavities suddenly increases and the fracture system becomes complicated, revealing that the material has entered the stage of fatigue cumulative damage. This nonlinear response is closely related to the fatigue limit characteristics of the material; high-frequency energy input not only accelerates the connection of damage-induced cavities but also activates the grain boundary dislocation movement of multiple slip systems.

**Figure 10:**
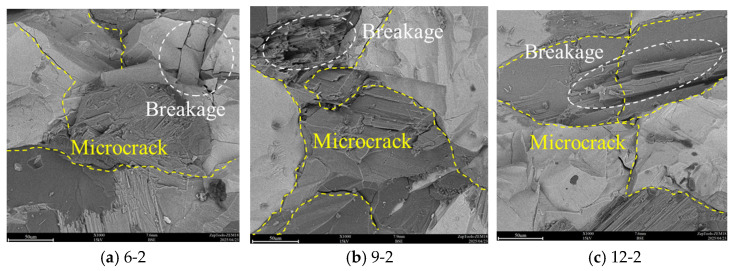

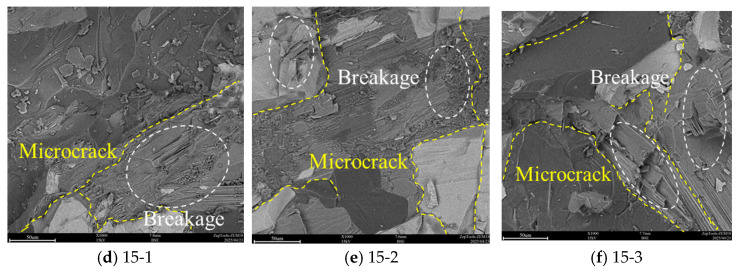
SEM scanning image of the pillar.

The SEM observation results of the pillar part reveal a distinct correlation between its failure characteristics and those of the backfill, as illustrated in Figure 10. For narrower pillars (6–9 mm), the backfill exhibits numerous damage-induced cavities and fractures, while the pillar itself undergoes relatively minor damage, with only a few fractures and localized fragmentation. This indicates that in the case of narrow pillars, most of the load is borne by the backfill, resulting in more severe damage to this area.

As the pillar width increases (12–15 mm), the damage to the pillar itself becomes more severe, with multiple breakage areas and more long fractures observed, while the damage to the backfill is alleviated. This change suggests that wider pillars can share more of the load, thereby reducing the burden on the backfill, but at the same time, the pillars themselves are subjected to greater stress. Under high-disturbance conditions (5 MPa), the pillars develop more complex fracture networks and more breakage areas, indicating that the repeated action of cyclic loading exacerbates the fatigue damage of the pillar material, leading to the gradual deterioration of its internal structure.

### 3.6. Quantitative Comparison of Mechanical Responses

To systematically assess the coupled effects of pillar width and disturbance amplitude, a quantitative comparison of key parameters is summarized as follows:

(1) Strength Enhancement With Pillar Width: When the disturbance amplitude is fixed at 3 MPa, the UCS increases from 14.96 MPa (6 mm) to 23.67 MPa (15 mm), an increase of 58.2%, while the elastic modulus increases from 1.89 GPa to 2.61 GPa, an increase of 38.1%.
(2) Strength Deterioration Under Higher Disturbance: For the 6 mm wide specimens, increasing the disturbance amplitude from 3 MPa to 5 MPa reduces UCS by 30.9% (from 14.96 MPa to 10.34 MPa). For the 15 mm wide specimens, the same amplitude increase causes only a 4.8% reduction, demonstrating the superior cyclic stability of wider pillars.
(3) Acoustic Emission Characteristics: Under a disturbance amplitude of 4 MPa, the cumulative ringdown counts at failure increase from 119,564 (6 mm) to 189,341 (15 mm), an increase of 58.3%, indicating that wider pillars undergo more extensive microcracking before failure.
(4) Microscopic Damage Features: SEM observations show that when pillar width increases from 6 mm to 15 mm under 4 MPa disturbance, the number of damage-induced cavities in the backfill decreases by approximately 62%, reflecting reduced localized damage concentration.

## 4. Conclusions

In this study, the mechanical properties and crack evolution mechanism of pillar–backfill composite materials under cyclic loading were investigated using an indoor cyclic loading test system. The main conclusions are as follows:

(1) Pillar width is a key parameter affecting the mechanical properties of PBCM. Samples with wider pillars (12–15 mm) exhibit superior bearing performance, with their uniaxial compressive strength and elastic modulus increasing by 88.6% and 42.7% respectively compared to those with narrower pillars (6–9 mm). It is noteworthy that samples with wider pillars show significantly lower sensitivity to disturbance amplitude; for instance, the UCS of 15 mm pillars only decreases by 4.8% under a 5 MPa disturbance, demonstrating better cyclic stability.
(2) Samples with narrow pillars accumulate damage more rapidly under cyclic loading, characterized by frequent high-energy acoustic emission events, and shear cracks account for over 50% under a 5 MPa disturbance. In contrast, samples with wide pillars exhibit more uniform damage distribution and stronger anti-disturbance capabilities, with their strain field evolution showing progressive characteristics.
(3) Observations of macroscopic failure modes indicate that pillar width directly influences the failure pattern. Samples with narrow pillars mainly undergo tension-dominated failure, where cracks mostly originate from interfaces and propagate rapidly. Samples with wide pillars, however, present shear-tension composite failure with more complex crack propagation paths. Particularly under high-disturbance (5 MPa) conditions, wide pillars can still maintain partial integrity, showing obvious characteristics of progressive failure.
(4) Given that the present study was conducted on small-scale composite specimens, future work will include large-scale specimens and field-cored samples to further validate the engineering applicability of the findings.

## Figures and Tables

**Table 1:** Experimental program for low-frequency perturbations.

Sample Name	Pillar Width/mm	Amplitude/ MPa	Disturbance Initial Value *σ_m_*/MPa	Disturbance Frequency/ Hz	Number of Cycles
6-1	6 mm	3 MPa	6.6 MPa	5 Hz	1000
6-2		4 MPa			
6-3		5 MPa			
9-1	9 mm	3 MPa	7.5 MPa		
9-2		4 MPa			
9-3		5 MPa			
12-1	12 mm	3 MPa	8.5 MPa		
12-2		4 MPa			
12-3		5 MPa			
15-1	15 mm	3 MPa	10.2 MPa		
15-2		4 MPa			
15-3		5 MPa			

**Table 2:** The UCS and E values of the samples.

Sample Name	UCS/MPa	E/GPa
6-1	14.96	1.89
6-2	12.15	1.78
6-3	10.34	1.71
9-1	16.52	2.09
9-2	15.65	1.91
9-3	13.11	1.83
12-1	18.92	2.23
12-2	17.36	2.20
12-3	17.21	2.16
15-1	23.67	2.61
15-2	22.92	2.54
15-3	22.53	2.53

## Data Availability

The original contributions presented in this study are included in the article. Further inquiries can be directed to the corresponding author.

## Abbreviations

The following abbreviations are used in this manuscript:

PBCM	Pillar–Backfill Composite Materials
AE	Acoustic emission
DIC	Digital image correlation

## References

[1] Gao T., Sun W., Liu Z., Cheng H.Y. (2022). Investigation on fracture characteristics and failure pattern of inclined layered cemented tailings backfill. Constr. Build. Mater..

[2] Qiu H.F., Zhang F.S., Liu L., Huan C., Hou D.Z., Kang W. (2022). Experimental study on acoustic emission characteristics of cemented rock-tailings backfill. Constr. Build. Mater..

[3] Gaili X., Erol Y., Wang Y.D. (2023). Progress and prospects of mining with backfill in metal mines in China. Int. J. Miner. Metall. Mater..

[4] Cheng A.P., Zhou C.S., Huang S.B., Zhang Y.S., Pei M.S. (2022). Study on the nonlinear deformation characteristics and constitutive model of cemented tailings backfill considering compaction hardening and strain softening. J. Mater. Res. Technol..

[5] Zhang C., Guo J., Taheri A., Song W.D., Wang X.L., Xia W.H. (2024). Mechanical characteristics and constitutive model of cemented tailings backfill under temperature-time effects. J. Build. Eng..

[6] Zhang Y., Liu Y.Z., Lai X.P., Cao S.G., Yang Y.B., Yan B.X., Bai L.C., Tong L., He W. (2023). Transport mechanism and control technology of heavy metal ions in gangue backfill materials in short-wall block backfill mining. Sci. Total Environ..

[7] Hou Y.Q., Yin S.H., Yang S.X., Chen X., Du H.H. (2023). Mechanical properties, damage evolution and energy dissipation of cemented tailings backfill under impact loading. J. Build. Eng..

[8] Li Y., Fu J.X., Wang K., He Z.Q. (2024). Influence of shell ash on pore structure and mechanical characteristics of cemented tailings backfill. Constr. Build. Mater..

[9] Zhang X.Z., Wu D., Lu H., Liu L., Zheng S.L. (2023). Improvement of tailings gradation on workability and strength of cemented tailings backfill. Constr. Build. Mater..

[10] Song X.P., Huang Y.C., Wang S., Yu H.G., Hao Y.X. (2023). Macro-mesoscopic mechanical properties and damage progression of cemented tailings backfill under cyclic static load disturbance. Compos. Struct..

[11] Zhu T.Y., Chen Z.H., Wang Z.Y., Cao J., Hao J.S., Zhou Z.H. (2025). Experimental and DEM analyses of type I fracture characteristics of waste rock aggregate reinforced cemented tailing backfill. Theor. Appl. Fract. Mech..

[12] Gan D.Q., Sun H.K., Xue Z.L., Liu Z.Y., Yang X. (2023). Damage evolution and strength prediction model of soda residue modified cemented tailings backfill under uniaxial compression. Constr. Build. Mater..

[13] Wang X.L., Li Z.F., Guo J.P., Lu C.W., Jiang H.Q., Mei J.W. (2024). Experimental and numerical investigations on damage mechanical behaviors of surrounding rock-backfill composite under uniaxial compression. Constr. Build. Mater..

[14] Zhao K., Huang M., Zhou Y., Yan Y.J., Wan W.L., Ning F.J., He Z.W., Wang J.Q. (2022). Synergistic deformation in a combination of cemented paste backfill and rocks. Constr. Build. Mater..

[15] Cui B.Q., Feng G.R., Bai J.W., Xue G.L., Wang K., Shi X.D., Wang S.Y., Wang Z.H., Guo J. (2023). Failure characteristics and the damage evolution of a composite bearing structure in pillar-side cemented paste backfilling. Int. J. Miner. Metall. Mater..

[16] Xia K.Z., Chen C.X., Liu X.T., Liu X.M., Yuan J.H., Dang S. (2023). Assessing the stability of high-level pillars in deeply-buried metal mines stabilized using cemented backfill. Int. J. Rock Mech. Min. Sci..

[17] Wu S.C., Li T.L., Cheng H.Y., Zhang X.Q., Zhao Z.Q. (2021). Mechanical response and stability of horizontal pillar size evolution inhigh stress environment. J. Cent. South Univ..

[18] Sherizadeh T., Kulatilake P.H.S.W. (2016). Assessment of roof stability in a room and pillar coal mine in the U.S. using three-dimensional distinct element method. Tunn. Undergr. Space Technol..

[19] Liu Y., He B., Dai F., Zhang Q., Liu Y. (2024). Mechanical responses of chemically corroded sandstone under cyclic disturbance: Insights from fatigue properties and macro-micro fracturing mechanism. Int. J. Rock Mech. Min. Sci..

[20] Xu J., Xiao X.C., Ma L., Luo S., Jin J.X., Wu B.J. (2024). Experimental study of the damage characteristics of rocks containing non-penetrating cracks under cyclic loading. Int. J. Min. Sci. Technol..

[21] Long D.Y., Wang Y., Li C.H., Wu Y.F. (2024). Macro-meso fatigue fracture and instability of rock-backfill composite structure under increasing-amplitude cyclic loading. Constr. Build. Mater..

[22] Zhou Y.Q., Sheng Q., Li N., Fu X.D. (2021). The relationship between dynamic strength and strain rate and damage to rock materials subjected to dynamic cyclic loading. Geomech. Geophys. Geo-Energy Geo-Resour..

[23] Li J.T., Shi Z.M., Yuan F.G., Guo B.S., Wang M.X., Lin H., Han D.Y., Li K.H. (2025). Mechanical properties and fracture damage behavior of thermal storage rocks under constant amplitude low cycle fatigue loading. Eng. Fract. Mech..

[24] Gan D.Q., Lu Y.Z., Sun H.K., Liu Z.Y., Zhang Y.J. (2024). Mechanical response and damage constitutive model of early-age cemented paste backfill after cyclic loading. J. Build. Eng..

[25] Wang L.J., Luan H.J., Jiang Y.J., Wang C.S., Zhang S.H., Li X.P., Wei J.L. (2025). Study on mechanical properties and damage evolution of pillar-backfill composite under cyclic loading. Constr. Build. Mater..

[26] Liang B., Wang D., Jiang Y.J., Luan H.J., Liu J.K., Wang J.L. (2024). Analysis of fracture modes and acoustic emission characteristics of low-frequency disturbed coal rock bodies with different cyclic amplitudes. Fatigue Fract. Eng. Mater. Struct..

[27] Zhou W., Cheng J.L., Zhang G.K., Li H.B., Cheng Y.G., Ma G., Ji X. (2021). Effects of Wetting–Drying Cycles on the Breakage Characteristics of Slate Rock Grains. Rock Mech. Rock Eng..

[28] Li C.X., Liu Y.S., Li L.S., Wang Z.H., Li H. (2025). Texture-Based Segmentation of SEM Images of Shale Rocks and Estimation of Meso-Scale Elastic Modulus by 2D FEM. Rock Mech. Rock Eng..

[29] Shan T.C., Li Z.H., Zhang X., Wang X.R., Jia H.S., Wang E.Y., Zhang Q.C., Niu Y., Wang D.M. (2025). Superstatistical approach of electric potential and acoustic emission for investigating damage evolution and precursor of water-bearing sandstone under uniaxial compression. Int. J. Rock Mech. Min. Sci..

